# FGFR3 expression in primary and metastatic urothelial carcinoma of the bladder

**DOI:** 10.1002/cam4.262

**Published:** 2014-05-21

**Authors:** Elizabeth A Guancial, Lillian Werner, Joaquim Bellmunt, Aristotle Bamias, Toni K Choueiri, Robert Ross, Fabio A Schutz, Rachel S Park, Robert J O'Brien, Michelle S Hirsch, Justine A Barletta, David M Berman, Rosina Lis, Massimo Loda, Edward C Stack, Levi A Garraway, Markus Riester, Franziska Michor, Philip W Kantoff, Jonathan E Rosenberg

**Affiliations:** 1 Dana-Farber Cancer InstituteBoston, Massachusetts; 2 University of RochesterRochester, New York; 3 Biostatistics and Computational Biology, Harvard Medical SchoolBoston, Massachusetts; 4 Htal De Mar Research Institute-IMIMBarcelona, Spain; 5 Department of Clinical Therapeutics, University of Athens Medical SchoolAthens, Greece; 6 Department of Pathology, Brigham and Women's Hospital, Harvard Medical SchoolBoston, Massachusetts; 7 Department of Pathology and Molecular Medicine, Queen's UniversityKingston, Ontario, Canada; 8 Center for Molecular Oncologic Pathology, Brigham and Women's HospitalBoston, Massachusetts; 9 Memorial-Sloan Kettering Cancer CenterNew York, New York

**Keywords:** Biomarker, bladder cancer, FGFR3, metastatic urothelial carcinoma, muscle-invasive urothelial carcinoma, targeted therapy

## Abstract

While fibroblast growth factor receptor 3 (FGFR3) is frequently mutated or overexpressed in nonmuscle-invasive urothelial carcinoma (UC), the prevalence of FGFR3 protein expression and mutation remains unknown in muscle-invasive disease. FGFR3 protein and mRNA expression, mutational status, and copy number variation were retrospectively analyzed in 231 patients with formalin-fixed paraffin-embedded primary UCs, 33 metastases, and 14 paired primary and metastatic tumors using the following methods: immunohistochemistry, NanoString nCounterTM, OncoMap or Affymetrix OncoScanTM array, and Gain and Loss of Analysis of DNA and Genomic Identification of Significant Targets in Cancer software. FGFR3 immunohistochemistry staining was present in 29% of primary UCs and 49% of metastases and did not impact overall survival (*P* = 0.89, primary tumors; *P* = 0.78, metastases). FGFR3 mutations were observed in 2% of primary tumors and 9% of metastases. Mutant tumors expressed higher levels of FGFR3 mRNA than wild-type tumors (*P* < 0.001). FGFR3 copy number gain and loss were rare events in primary and metastatic tumors (0.8% each; 3.0% and 12.3%, respectively). FGFR3 immunohistochemistry staining is present in one third of primary muscle-invasive UCs and half of metastases, while FGFR3 mutations and copy number changes are relatively uncommon.

## Introduction

The treatment of metastatic urothelial carcinoma (UC) of the bladder has not advanced significantly in over 20 years. Platinum-based combination chemotherapy remains the standard treatment for this disease, and no effective salvage therapies are FDA-approved in the United States. Understanding the biology of UC to identify new druggable targets is required to improve clinical outcomes.

The fibroblast growth factor (FGF) family of transmembrane tyrosine kinase receptors mediates proliferation in response to FGF stimulation and has been implicated in the pathogenesis of UC. Fibroblast growth factor receptor 1 (FGFR1) is overexpressed in a subset of UC specimens, and some invasive UC cells lines are dependent on FGFR1 protein for proliferation 1.

## Materials and Methods

### Patients

Primary UC formalin-fixed paraffin-embedded (FFPE) bladder specimens from either transurethral resections or cystectomies were provided by the Hospital del Mar, Barcelona, Spain (*N* = 107) and the Hellenic Cooperative Oncology Group (HCOG), Athens, Greece (*N* = 110) under Institutional Review Board (IRB) approved protocols. Primary (*N* = 14) UC FFPE specimens from radical cystectomies and metastatic (*N* = 33) UC FFPE specimens from metastectomies were identified from the Brigham and Women's Hospital (BWH) Pathology Department, Boston, under IRB-approved protocols. All patients with primary UC tumors had muscle-invasive tumors and went on to develop metastatic UC. Paired primary and metastatic tumors were available for 14 patients who overlapped between the primary tumor and metastases cohorts. Normal bladder tissue (from cystectomies for nonmalignant indications) was obtained from the BWH with IRB approval. Two genitourinary pathologists reviewed the slides and identified tumor and normal tissue; D. B. reviewed tissue from the BWH and Spanish cohorts and J. B. reviewed tissue from the Greek cohort.

Tissue cores of 0.6 mm were taken from each specimen for DNA and total RNA extraction and tissue microarrays (TMA) construction. Three cores of each case were embedded in the tissue microarray, and normal urothelium cores from normal bladder tissue from non-cancer patients were included as controls. Genomic DNA was extracted from tumors with the QiaAmp DNA FFPE Tissue Kit (Quiagen, Valencia, CA) on the Quiagen Robot according to the manufacturers' instructions. Total RNA was extracted from tumors with the FFPE kit on the Beckman Coulter Biomek platform (Beckman Coulter, Beverly MA) according to the manufacturers' instructions.

### Immunohistochemistry

Tissue microarrays with primary and metastatic tumors and normal bladder controls were stained with a commercially available anti-FGFR3 antibody (Santa Cruz, Dallas, TX, clone B-9, SC-13121). Antigen retrieval was performed in ethylene-diamine-tetra-acetic acid buffer using a microwave set on high for 5 min, repeated three times. Following antigen retrieval, slides were transferred to a BioGenex i6000 (Fremont, CA) automated staining deck. Slides were rinsed in a phosphate-buffered saline tween wash for 15 min, incubated in a commercial peroxidase blocking solution (Dako, Carpinteria, CA) for 30 min, and then incubated with commercial protein block (Dako) for 20 min. The slides were then incubated with the primary antisera to FGFR3 at a dilution of 1:50 for 1 h. The primary antisera was visualized using a peroxidase-based detection kit (Dako Envision), following the manufacturers' instructions. The slides were counterstained with hematoxylin (BioGenex) and coverslipped.

Tissue microarrays cores for the four TMA's were evaluated by a single pathologist (R. L.). Stain in the tumor cells was designated as nuclear, cytoplasmic, and/or membranous. Intensity of the stain was scored as absent (negative), weak, weak–moderate, moderate, or strong based on previously reported scoring systems 2,3. The presence of any staining was considered positive. Immunohistochemistry staining was repeated when necessary to demonstrate what was judged to be uniform staining of the slide. Any uneven individual core staining or edge effect, and any cores exhibiting extreme electrocautery changes or insufficient tumor present were excluded from the scoring analysis. Any background stain in the stroma was used as baseline in determining the epithelial score. Of the three cores represented for each patient case in the TMA, actual cases had either 0, 1, 2, or 3 cores available for scoring (due to exclusionary issues stated above), and this is reflected in the raw data.

### mRNA analysis

Total RNA was extracted from tumor specimens following manufacturer protocols (Ambion RecoverAll, Life Technologies, Grand Island, NY). mRNA transcript expression of FGFR3 was quantified using color-coded oligonucleotides synthesized by NanoString nCounterTM gene expression system and hybridized to these transcripts, as has been previously described for FFPE archival samples 4. Transcripts were counted using the automated NanoString nCounter® system (NanoString Technologies, Seattle, WA). Counts were normalized with the nSolver Analysis Software (version 1.0) in which mRNA expression was compared to internal NanoString controls, several housekeeping genes (ACTB, GAPDH, HPRT1, LDHA, PFKP, PGAM1, STAT1, TUBA4A, VIM), and UC-invariant genes (ANGEL1, DDX19A, NAGA, RPS10, RPS16, RPS24, RPS29), which were identified by analyzing gene expression variances in several published datasets 5,6. Differential expression of FGFR3 mutants versus wild-type (WT) tumors was calculated with the edgeR package 7.

### FGFR3 mutation analysis

Two technologies were used to test specimens for FGFR3 mutations: OncoMap mass spectrometric genotyping based on the Sequenom MassARRAY® technology (Sequenom Inc., San Diego, CA) 8,9, and Affymetrix OncoScan FFE Express molecular inversion probe (MIP) arrays 10 (Table1). The OncoMap platform consists of two assays, iPLEX genotyping and multibase homogenous Mass-Extend (hME) extension chemistry; only mutations detected by both methods were considered “validated mutations” in OncoMap. For both OncoMap and MIP arrays, genomic DNA was quantified using Quant-iT PicoGreen dsDNA Assay Kit (Invitrogen, Grand Island, NY) per manufacturer's protocol.

**Table 1:** FGFR3 mutations included in assays.

FGFR3 mutations	ONCOMAP (versions 1 and 3)	MIP
Y241C	•	
R248C	•	•
S249C	•	•
H284fs^*^10	•	
E322K	•	
G370C	•	•
S371C		•
Y373C	•	•
F384L	•	
A391E	•	•
K650E	•	
K650Q	•	•
K650M	•	•
K650T	•	

Filled circles indicate in which of the assays (or both) the listed mutations are included.

OncoMap mass spectrometric genotyping based on the Sequenom MassARRAY® technology (Sequenom Inc.) was performed as previously described with some modifications 11. A total of 250 ng of DNA was used for mutation analysis. Probes were designed that enabled mutation detection. Hundred nanograms of tumor-derived genomic DNA was subjected to whole genome amplification (WGA). Next, up to 18-multiplexed PCR was performed on tumor genomic DNA to amplify regions harboring loci of interest. After denaturation, PCR products were incubated with the probes that anneal immediately adjacent to the query nucleotide. Mass spectrometric genotyping using iPLEX chemistries was performed (Sequenom Inc.) extending the probes with one base in the presence of chain-terminating di-deoxynucleotides that generate allele-specific DNA products. The extension products were spotted onto a specially designed chip and analyzed by matrix-assisted laser desorption/ionization time-of-flight (MALDI-TOF) mass spectrometry to determine the mutation status based on the difference in mass of the mutant and WT base.

Next, an automated mutation-calling algorithm was performed to identify candidate mutations. Putative mutations were further filtered by a manual review and selected for validation using multibase hME chemistry with a maximum pooling of six assays on the remaining 150 ng DNA of each sample. Primers and probes used for hME validation were designed using the Sequenom MassARRAY Assay Design 3.0 (Farmingdale, NY) software, applying default multibase extension parameters. OncoMap “validated mutations” were identified by both iPLEX and hME.

Affymetrix OncoScanTM FFPE Express MIP arrays were performed as previously reported 10. DNA from primary and metastatic tumors was hybridized to Affymetrix OncoScanTM FFPE Express 2.0 SNP MIP arrays. Median probe spacing was 9 kb. NEXUS software (Concord, OH) was used to estimate copy numbers.

### FGFR3 pathway copy number analysis

Normalized copy number data were segmented using Gain and Loss Analysis of DNA (GLAD) with default parameters available in GenePattern (version 3.3.3; Cambridge, MA) 12.

The Genomic Identification of Significant Targets in Cancer (GISTIC) (version 2.0.12; Cambridge, MA) software was used with a confidence level of 0.95 and otherwise default parameters to identify regions of the genome that were significantly amplified or deleted across a set of samples 13. High-level amplifications and deletions were identified using log base 2 ratio thresholds of 0.9 and <−0.3, respectively.

### Biostatistics

Patient and clinical characteristics were summarized as numbers and percentages. FGFR3 staining intensity was tabulated by category and summarized as numbers and percentages with 95% confidence intervals (CI). Exact binominal test was used to calculate 95% CI. Fisher's exact test was used to assess the associations of FGFR3 mutation and types of tumor. The median overall survival (OS) for the primary cohort was measured from the start of chemotherapy for metastatic disease. The median OS for the metastatic cohort was measured from the time of diagnosis of metastatic disease rather than initiation of chemotherapy because this clinical information was not available for all patients. However, when these data were available, the median time from diagnosis of metastases to the start of chemotherapy was 1.2 months, which suggested that the difference in OS as measured by these two definitions was not clinically significant. Kaplan–Meier estimate was used to summarize median OS. Cox regression model was used to assess the association of FGFR3 staining and OS and hazard ratio with 95% CI was presented. The association of OS and other measured parameters, such as FGFR3 mutation or copy number variation (CNV), was not performed because of limited sample sizes with the presence of these alterations.

## Results

### Patients and tumors

A total of 231 patients with primary tumor samples available were included in the primary cohort; 206 patients in this cohort had documentation of the development of metastatic disease (Table2). The metastasis cohort included 33 patients with metastatic tumor samples, for which clinical data were available for 31 patients.

**Table 2:** Patient clinical characteristics.

	*N* (%)
*Primary tumor cohort (N = 231)*	
Median OS^1^ (16 months)	
ECOG PS	
0	90 (39)
1 + 2	102 (78)
Missing	39 (17)
Visceral disease^3^	
No	113 (49)
Yes	87 (38)
Missing	31 (13)
ECOG PS >0 and presence of visceral disease	40 (17)
Survival	
Alive	77 (33)
Dead	136 (59)
Missing	18 (8)
Metastatic cohort (*N* = 31)	
Median OS^2^ (11 months)	
Metastatic site	
Visceral	16 (52)
Local	7 (23)
Lung	13 (42)
Lymph node	12 (39)
Liver	8 (26)
Bone	4 (13)
Other	9 (29)

1 Median OS measured from time of initiation of chemotherapy for metastatic disease to death.

2 Median OS measured from time of diagnosis of metastatic disease to death.

3 Visceral disease is defined as metastases to internal organs such as lung or liver but not lymph nodes.

### FGFR3 immunohistochemistry

**Figure 1:**
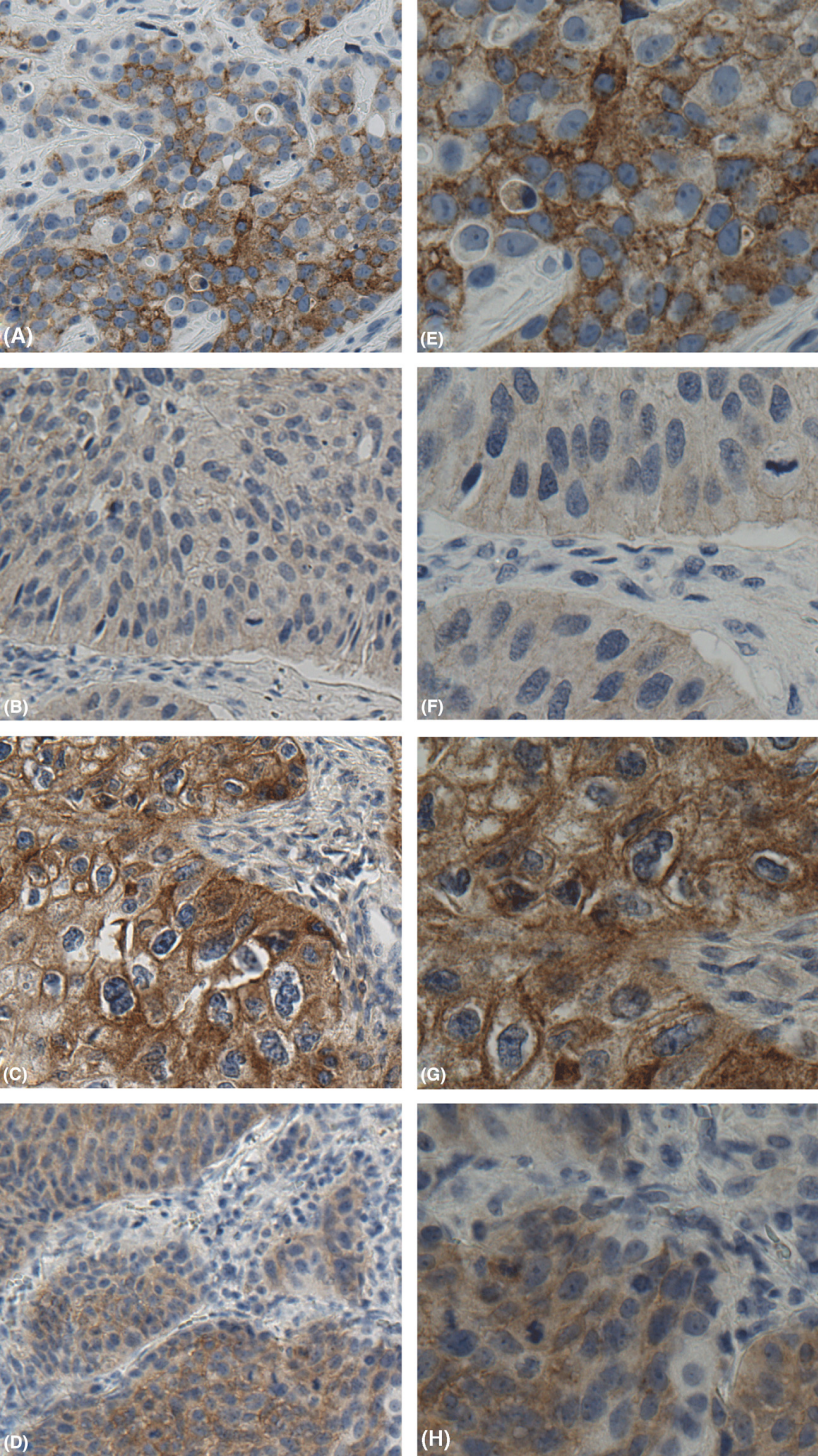
FGFR3 immunostaining in muscle-invasive UC of the bladder with metastatic phenotype. (A and E) Low- and high-power (20× and 40×) FGFR3 expression in invasive urothelial carcinoma, graded as moderate with both cytoplasmic and membranous staining. (B and F) Low- and high-power (20× and 40×) FGFR3 expression in metastatic urothelial carcinoma, graded as weak with cytoplasmic staining evident. (C and G) Low- and high-power (20× and 40×) FGFR3 expression in metastatic urothelial carcinoma, graded as strong, with prominent cytoplasmic and membranous staining. (D and H) Low- and high-power (20× and 40×) FGFR3 expression in metastatic urothelial carcinoma, graded as moderate, with prominent cytoplasmic staining.

**Figure 2:**
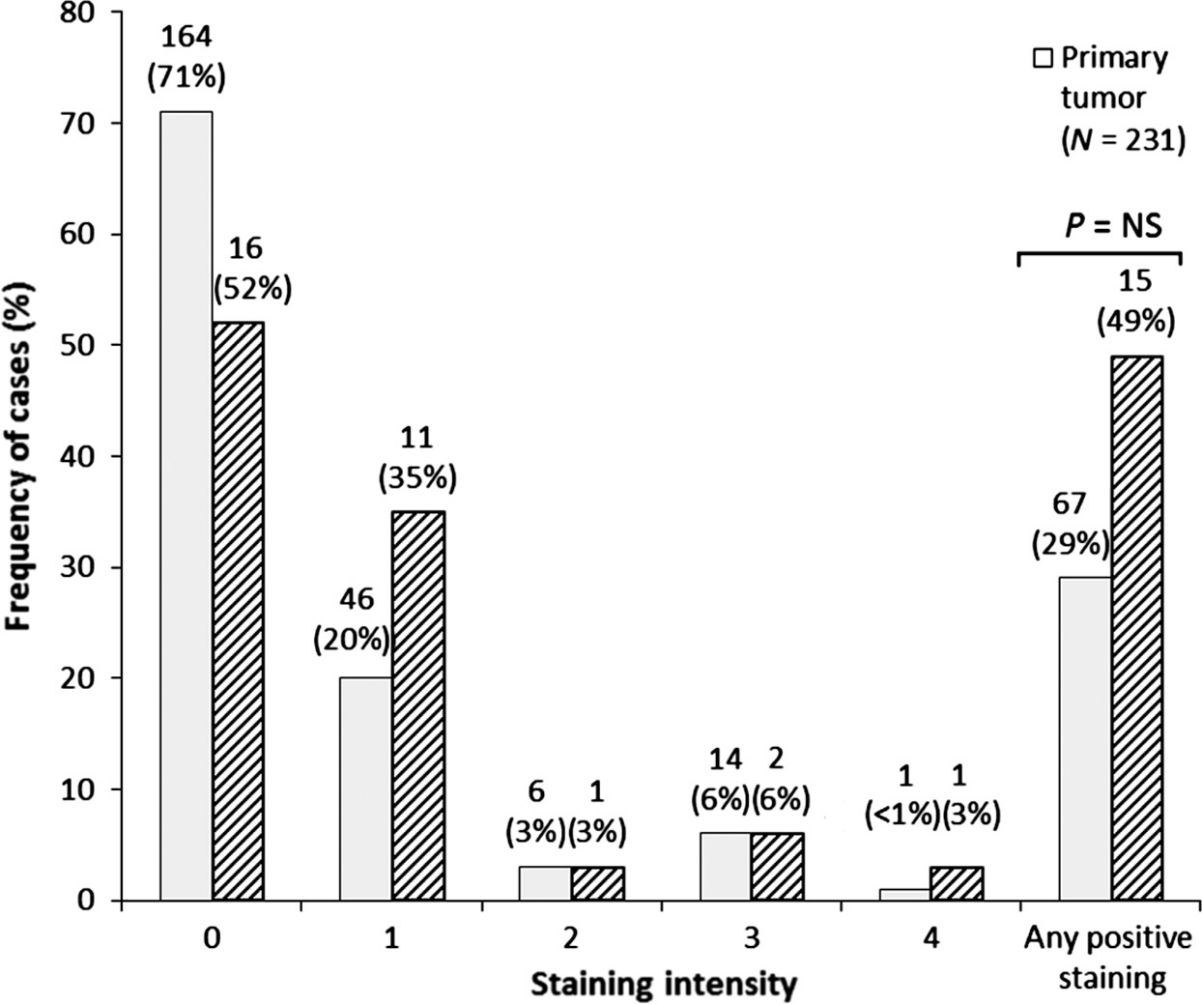
Immunohistochemical staining of FGFR3 in primary and metastatic tumors. Negative staining = 0, weak = 1, weak–moderate = 2, moderate = 3, strong = 4. *P = NS by Fisher's exact test (two tailed) between negative and any positive staining.

Immunohistochemical staining data were evaluable from 231 primary tumors and 31 metastases (Fig.1). Positive staining for FGFR3 was detected in 29% (95% CI = [23–35%]) of primary tumors and 49% (95% CI = [30–67]) of metastatic tumors (Fig.2). Less than 1% of primary tumors and 3% of metastases had strong staining. There is no statistically significant difference in the percentage staining between primary tumors and metastases (41% vs. 49%, respectively). Of 14 paired primary and metastatic tumors, three pairs were positive for FGFR3 staining, whereas seven pairs were negative for FGFR3 staining. Among primary tumors with positive staining, 60% of paired metastases were also positive.

**Figure 3:**
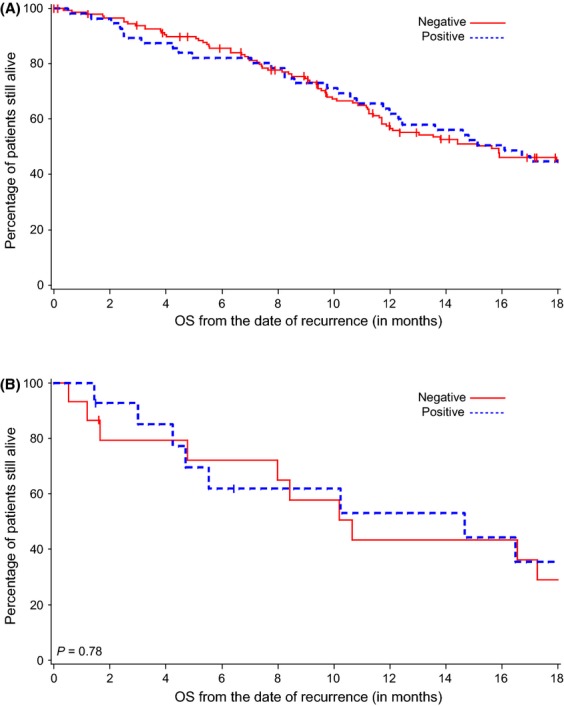
OS and FGFR3 staining in primary and metastatic tumors. (A) FGFR3 staining was not associated with a difference in OS in the primary tumor cohort (*P *=* *0.89). (B) FGFR3 staining was not associated with differences in OS from time of disease recurrence in the metastatic tumor cohort (*P *=* *0.78).

FGFR3 staining in the primary tumor was not associated with OS (HR = 1.03, 95% CI [0.7–1.50], *P* = 0.89) in patients who developed metastatic disease (*N* = 206) (Fig.3A). In metastatic tumor specimens (*N* = 31), FGFR3 staining did not correlate with OS (HR = 1.12, 95% CI [0.49–2.58], *P* = 0.78) (Fig.3B). Due to limited sample size, we were unable to determine whether specific subcellular locations of FGFR3 immunostaining were associated with FGFR3 expression in metastases or with OS.

### FGFR3 mRNA analysis

**Figure 4:**
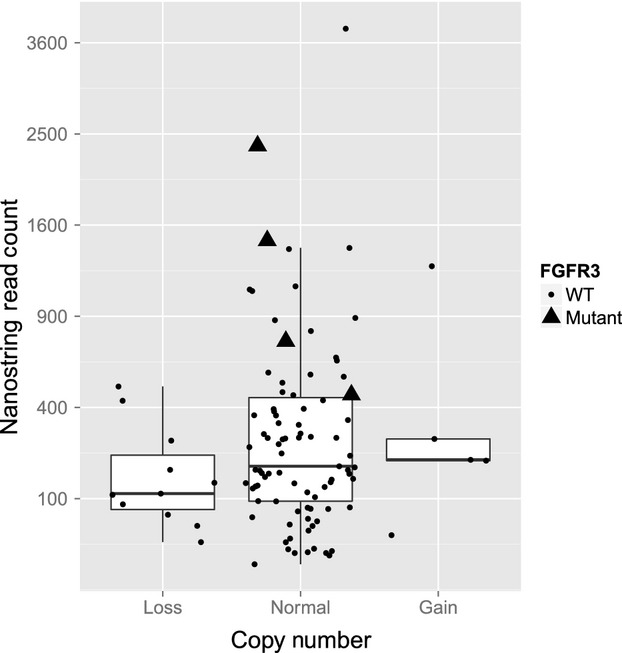
FGFR3 copy number and mRNA expression for FGFR3 mutant and wild-type (WT) tumors. The Nanostring read counts for all patients with loss, gain, or normal FGFR3 copy numbers are visualized with dots for WT tumors and triangles for FGFR3 mutants. In addition, the distributions of read counts in the three categories are visualized with box plots.

Tumors with FGFR3 mutations had a statistically significant increase in FGFR3 mRNA expression compared with WT tumors (*P* < 0.001) (Fig.4). One of two FGFR3-mutant tumors for which both mRNA and IHC data were available had weak FGFR3 protein expression by IHC; the other tumor had negative FGFR3 expression.

### FGFR3 mutation

Primary UC specimens (*N* = 131) were tested for FGFR3 mutations with either OncoMap- (version 1 and version 3) targeted hotspot sequencing or Affymetrix OncoScanTM FFPE Express MIP arrays (*N* = 30). A subset of eight primary tumors underwent mutation testing with both platforms. Metastatic UC specimens from Dana-Farber Cancer Institute (*N* = 33) were tested for FGFR3 mutations using Affymetrix MIP arrays.

There was no significant difference in the frequency of FGFR3 mutations between primary tumors (2%) and metastatic tumors (9%) (Table3) (*P* = 0.1). One primary tumor tested with both OncoMap and OncoScan demonstrated evidence of Y373C mutation using OncoMap, but the overall mutation frequency did not meet the threshold in OncoScan for a positive mutation. The remaining seven samples tested using both OncoMap and OncoScan were negative for mutations in each platform. None of the three metastases with Y373C mutations had paired primaries, although one had a paired normal bladder specimen that did not demonstrate evidence of Y373C mutation. No mutations were detected in any of the paired primary and metastatic specimens. Two of three metastases with FGFR3 mutations demonstrated positive FGFR3 expression by IHC: one had moderate staining and the second had strong staining.

**Table 3:** Frequency and type of FGFR3 mutations in primary tumors and metastases.

	FGFR3 mutations observed: *N*	FGFR3 mutant% (95% CI)	*P*
Primary tumor (*N* = 161)	R248C: 2 Y373C: 2	2% (1–6%)	0.1
Metastasis (*N* = 33)	Y373C: 3	9% (2–24%)	

### FGFR3 copy number analysis

Normal bladder specimens demonstrated no CNVs in FGFR3 (Table4). FGFR3 copy number gain and loss were rare events in primary tumors (0.8% each). Copy number gain and loss was observed in 3.0% and 12.1% of metastases, respectively. Co-occurrence of mutation and copy number gain of FGFR3 was observed in one specimen. Copy number gains or losses detected among metastatic samples were not seen in the primary tumor when available.

**Table 4:** DNA copy number variation of FGFR3 by tissue type.

Tissue	FGFR3 gain	FGFR3 loss
Normal bladder tissue (*N* = 37)	0	0
Primary tumor (*N* = 121)	1 (0.8%)	1 (0.8%)
Metastasis (*N* = 33)	1 (3.0%)	4 (12.1%)
FGFR3 mutant (*N* = 6)	1 (16.7%)	0

## Discussion

Our results demonstrate that some FGFR3 IHC staining is present in nearly one third of primary muscle-invasive UC tumors and half of metastases. While the sample size was relatively small, over half of metastases from paired primary tumors with FGFR3 expression retain FGFR3 protein expression, albeit at low levels in most specimens. Tissue cores were obtained from pathologist-reviewed H&E tumor specimens and incorporated into a TMA. Since the whole tissue section was not available to be stained for FGFR3, we do not know if FGFR3 expression is homogenous throughout the tumor. The presence or absence of FGFR3 IHC staining does not appear to have an impact on OS in patients with metastatic disease who are treated with platinum-based chemotherapy. On the basis of our findings, we believe that the importance of FGFR3 as a driver of tumor growth and progression in metastatic UC is unclear, and further functional evidence is required to support this hypothesis.

The frequency of FGFR3 mutation in our cohorts is low, with 2% in primary tumors and 9% in metastases, although the sample size was small and the confidence intervals are wide. A recent study by Gust et al. reported FGFR3 mutations in 11% (10/95) of cases of high-grade invasive UC 14. Ross et al. also recently published their finding of FGFR3 mutations in 6% (2/35) cases of high-grade metastatic muscle-invasive disease, both of which were detected in primary tumors 15. In contrast, previous reports have found FGFR3 mutations in up to 20% of muscle-invasive UC using mass spectrometry-based genotyping assay and 15% using sequencing 16,3. While Al-Ahmadie et al. used frozen tumors, the majority of which were from cystectomy cases for their mutation analysis, Tomlinson et al. performed their studies on FFPE tissue, although they did not specify whether these were TURBT or cystectomy samples. Thus, the use of FFPE tissue in our study is unlikely to be the reason for the lower mutation rate among our samples. Unlike Al-Ahmadie et al., we did not perform Sanger sequencing of all FGFR3 coding exons. However, the mutations identified in their study and in the work by Tomlinson et al. were included in our OncoMap and MIP platforms. The majority of our samples came from patients in Greece and Spain, raising the possibility that differences in exposures between these populations and those studied by Al-Ahmadie et al. and Tomlinson et al. may account for the differences in the mutation profile of these cohorts.

The most frequently identified FGFR3 mutation in our study was Y373C, followed by R248C. Other studies have reported S249C as the most commonly identified FGFR3 mutation, with lower frequencies of Y375C and R248C (10–20%) 3,17–19. The majority of patients in these studies had low-grade, nonmuscle-invasive disease in contrast to our muscle-invasive cohort. Thus, changes in tumor biology reflected by differences in grade or stage may account for some of this discrepancy. Although we only identified four FGFR3 mutants among 161 primary UC tumors and three mutants among 33 metastases, tumors with FGFR3 mutations in our study showed a statistically significant increase in FGFR3 mRNA levels and a trend toward increased FGFR3 protein expression by IHC, similar to what has been observed in nonmuscle-invasive disease 20,3. This preliminary finding of an association between FGFR3 mutation and protein expression requires confirmation in a larger cohort of tumors with FGFR3 mutations.

There are several limitations of this study. First, mutation analysis of FGFR3 was performed by hotspot sequencing using two different technologies, OncoMap and MIP array, rather than whole exon sequencing based on when the work was performed and technology available at that time; older samples were tested using Oncomap, and OncoScan was used when more recent samples became available. Among the eight tumors tested for mutations using both platforms, one tumor had a discordant result with Y373C mutation detected using OncoMap (iPLEX and hME) but the mutation, while detected in OncoScan, did not reach the frequency threshold for positivity. This discordance may reflect low tumor purity, heterogeneity, or subclonality within the tumor specimen. Since representative tissue cores were obtained from pathologist-reviewed tumor specimens and incorporated into a TMA, our results do not rule out heterogeneous staining and/or mutation profiles within tumors. In addition, no external validation of our results was possible, as no other clinically annotated cohort of patients with primary or metastatic UC was available for analysis.

It is important to note is that no established cutoffs have been developed for FGFR3 IHC staining. We reported any staining as “positive” based on a previous report 3, although many of our specimens showed weak staining. In contrast, other studies required positive FGFR3 staining in at least 5% of cells for a specimen to be considered positive 17,21. Despite using the same threshold for positivity, though, these groups reported different percentages of positive staining tumors ranging from 15% of pT2 and 2% of pT3 UC (all of which were moderately or poorly differentiated) 17 to 49.2% of high-grade pT2 UC 18.

Thus, despite a more inclusive threshold for positive FGFR3 staining, our observation that 29% of the primary tumors in our cohort had positive FGFR3 staining is reasonable based on the published literature. Finally, the majority of patients in our cohorts received platinum-based combination chemotherapy and thus their tumors may not be representative of the biology of UC in patients unable to receive chemotherapy due to impaired performance status.

Several lines of preclinical data suggest that FGFRs hold promise as therapeutic targets in UC. FGFR3 and FGFR1 are either mutated or overexpressed in the majority of nonmuscle-invasive UC 22, and in a percentage of muscle-invasive UC 17,23. Furthermore, inhibition of FGF signaling in both FGFR3 mutant and WT cell lines results in reduced proliferation, thus suggesting a role for FGFR3 “oncogene addiction” that is independent of mutation status 24,25. However, the clinical results to date have been disappointing. A phase II study of TKI258 (dovitinib), an oral inhibitor of FGFR3, VEGFR, and PDGFR, was recently terminated due to limited single agent activity in pretreated, advanced UC patients (NCT00790426), including in the cohort with a presumed FGFR3 mutation, despite encouraging in vitro inhibition of tumor proliferation 26. Other agents currently in development that may demonstrate increased clinical activity include pan-FGF inhibitors, dual FGFR, and VEGFR inhibitors and FGFR-specific antibodies conjugated to the gelonin toxin 27. The discrepancy between promising in vitro studies and lack of clinical benefit may reflect either lack of target specificity of the drug, that is, the wrong drug for the right target, or a lack of understanding of how FGFRs contribute to UC, which in turn prevents identification of a sensitive subset of tumors. Recent work demonstrates that FGFR3 fusion with transforming acid coiled coil 3 (TACC3) via 4p16.3 and t(4:7) rearrangements result in receptor activation independent of FGFR3 mutation or overexpression and confer sensitivity to FGFR-selective agents 28. This work suggests that chromosomal translocations, rather than mutational status or expression alone, could be predictive of response to FGFR inhibition. Furthermore, these fusions were identified in less than 10% of lines studied (4 of 43 and 2 of 32, respectively) 28. FGFR3 fusions (FGFR3-TACC3 and FGFR3-JAKMIP1) were recently identified in 5.7% (2/35) cases of high-grade metastatic muscle-invasive disease 15. Thus, small unselected phase II studies are unlikely to include patients whose tumors may be driven by these translocations.

FGFR3 IHC staining does not appear to have prognostic or predictive value in patients with metastatic UC. This finding, however, does not eliminate FGFR3 as a potential therapeutic target in metastatic disease. While FGFR3 staining is rarely intense, the presence of detectable protein in primary and metastatic tumors suggests that FGFR3 might be targeted by an antibody-mediated approach and result in successful growth inhibition, as recently demonstrated by Gust et al., provided we have predictive biomarkers with which to identify tumors that are dependent on FGFR3 signaling 14.

In summary, FGFR3 expression is present in both primary and metastatic UC, and the majority of metastases retain IHC expression of FGFR3 if it was expressed in the primary tumor. While FGFR3 mutation or copy number gain is a rare event, studies of these tumors may shed light on how FGFR3 signaling contributes to UC growth and proliferation. Furthermore, based on preclinical studies that suggest the FGFR pathway, and specifically FGFR3, may be a valid therapeutic target, we believe this area of investigation warrants further exploration.
